# Supplementary figures and images for: Construction of engineered RuBisCO Kluyveromyces marxianus for a dual microbial bioethanol production system
Source: PLoS One. 2021 Mar 4;16(3):e0247135. doi: 10.1371/journal.pone.0247135 (PMC7932148; doi:10.1371/journal.pone.0247135)

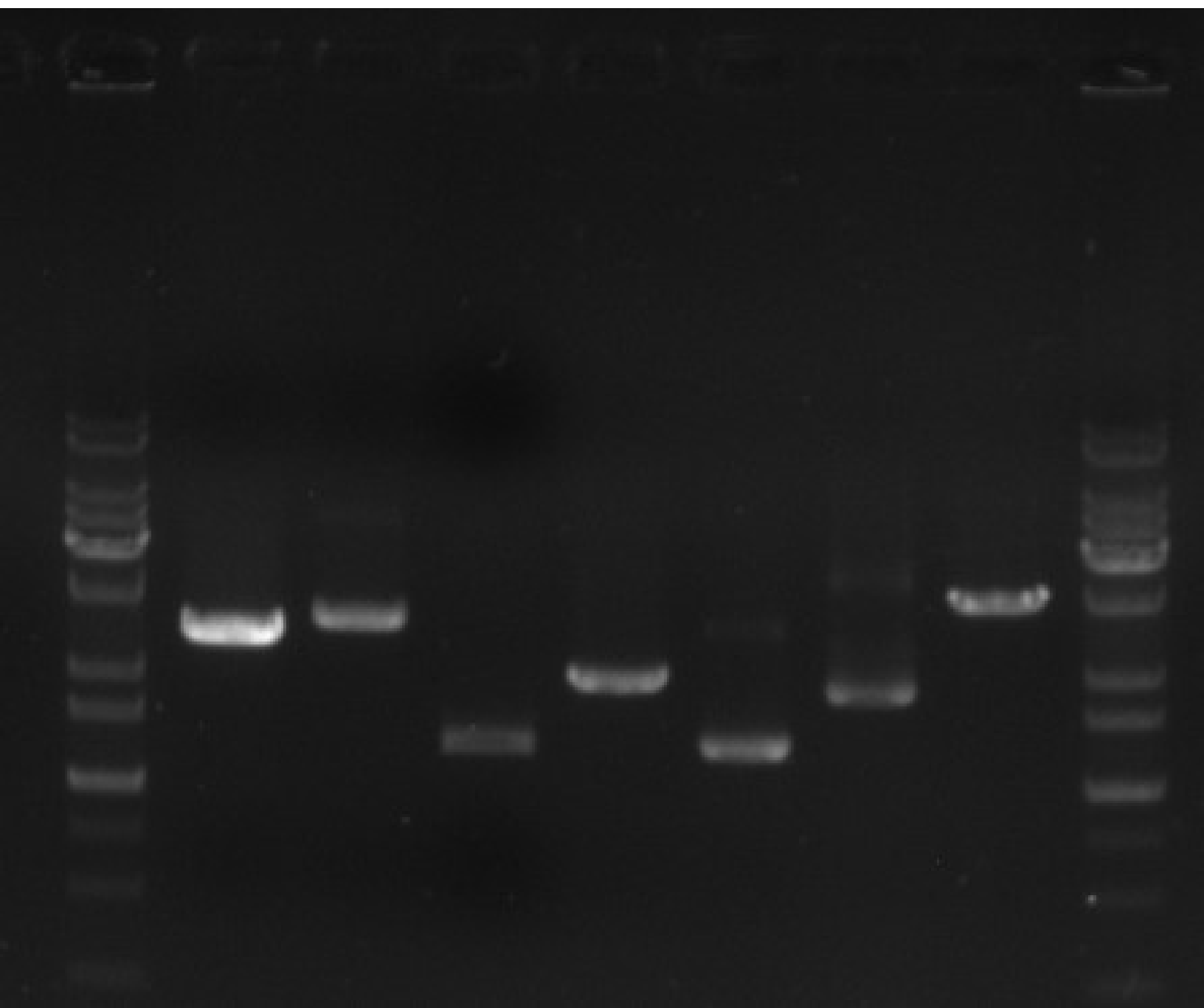

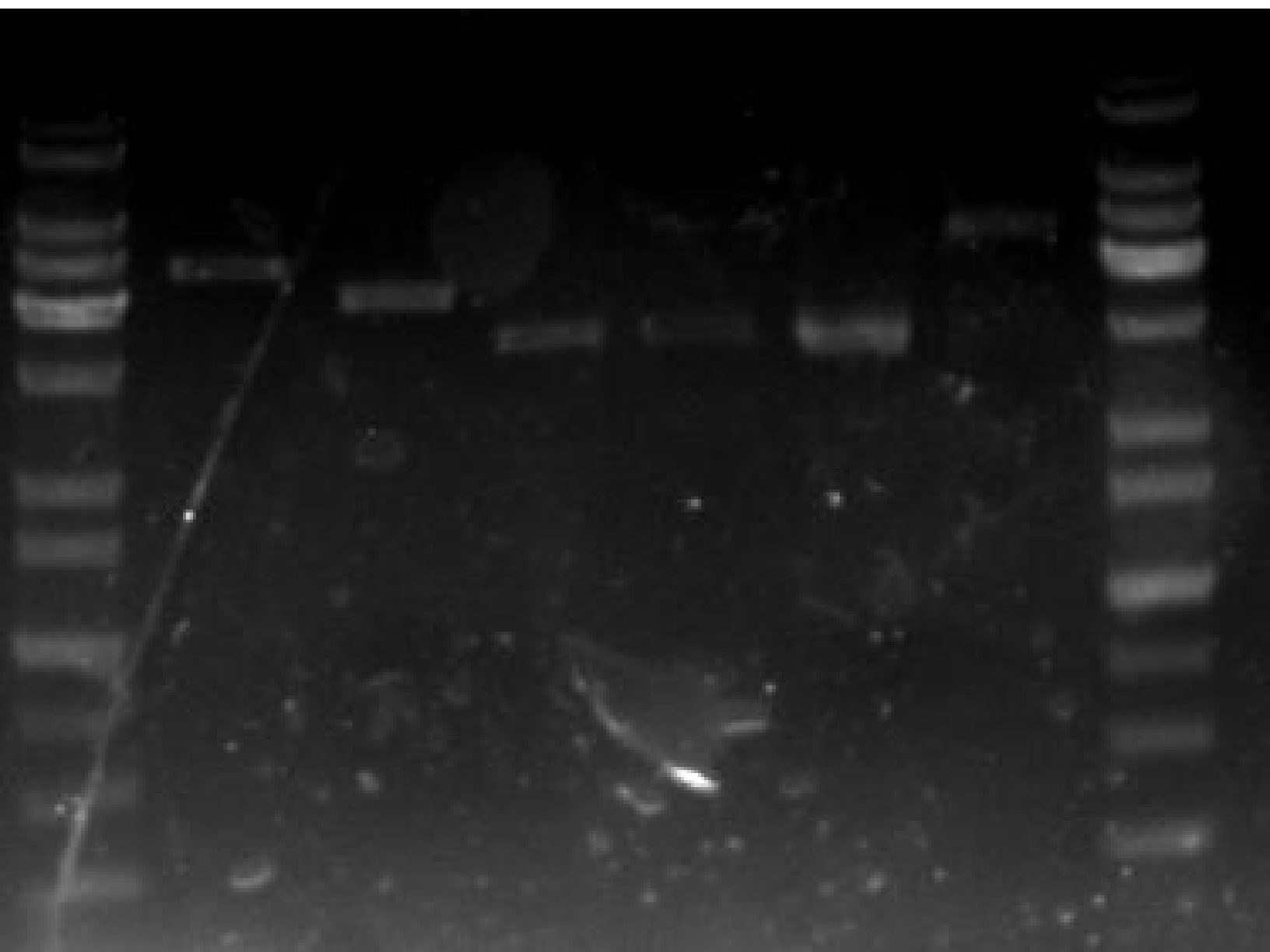

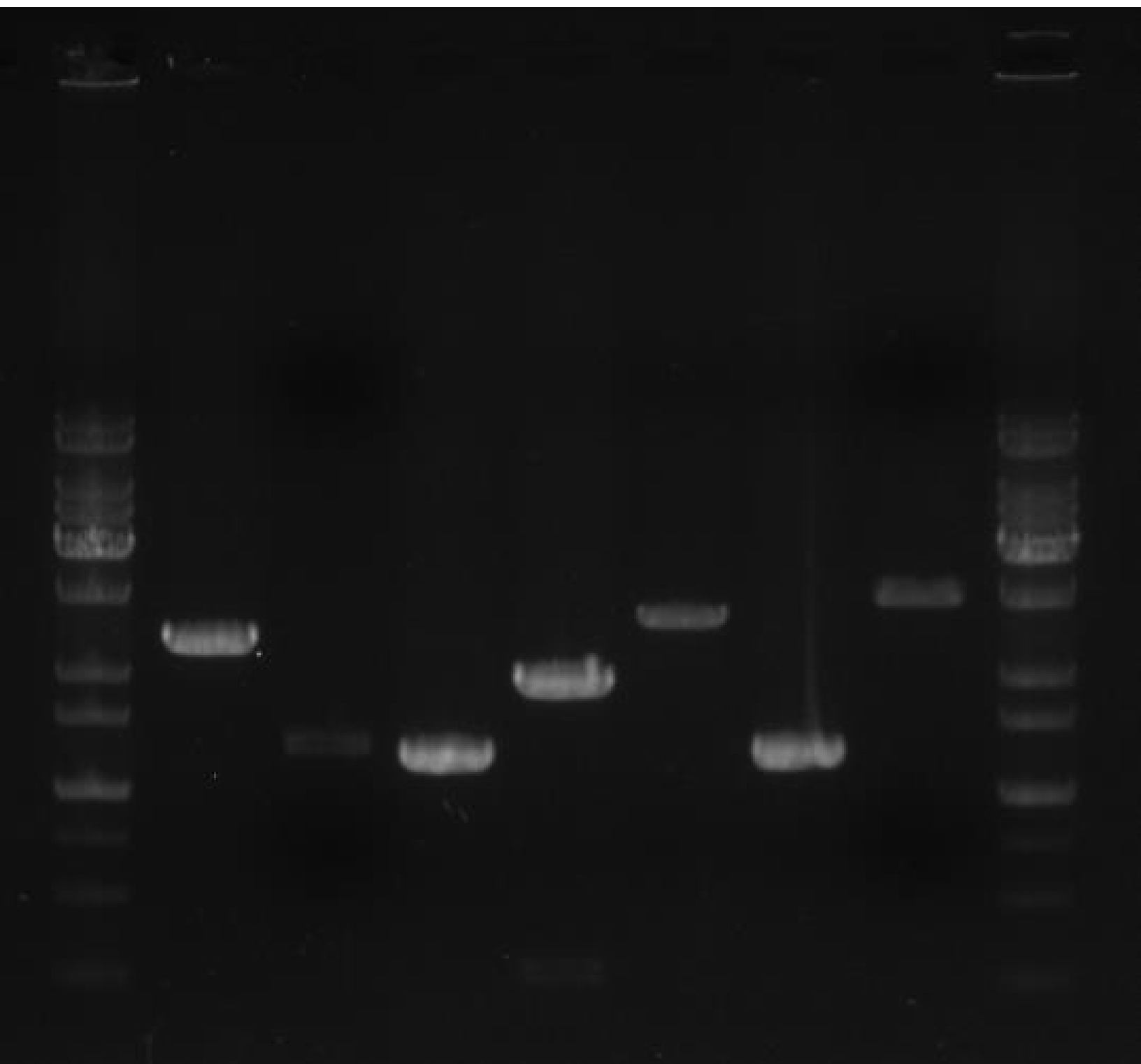

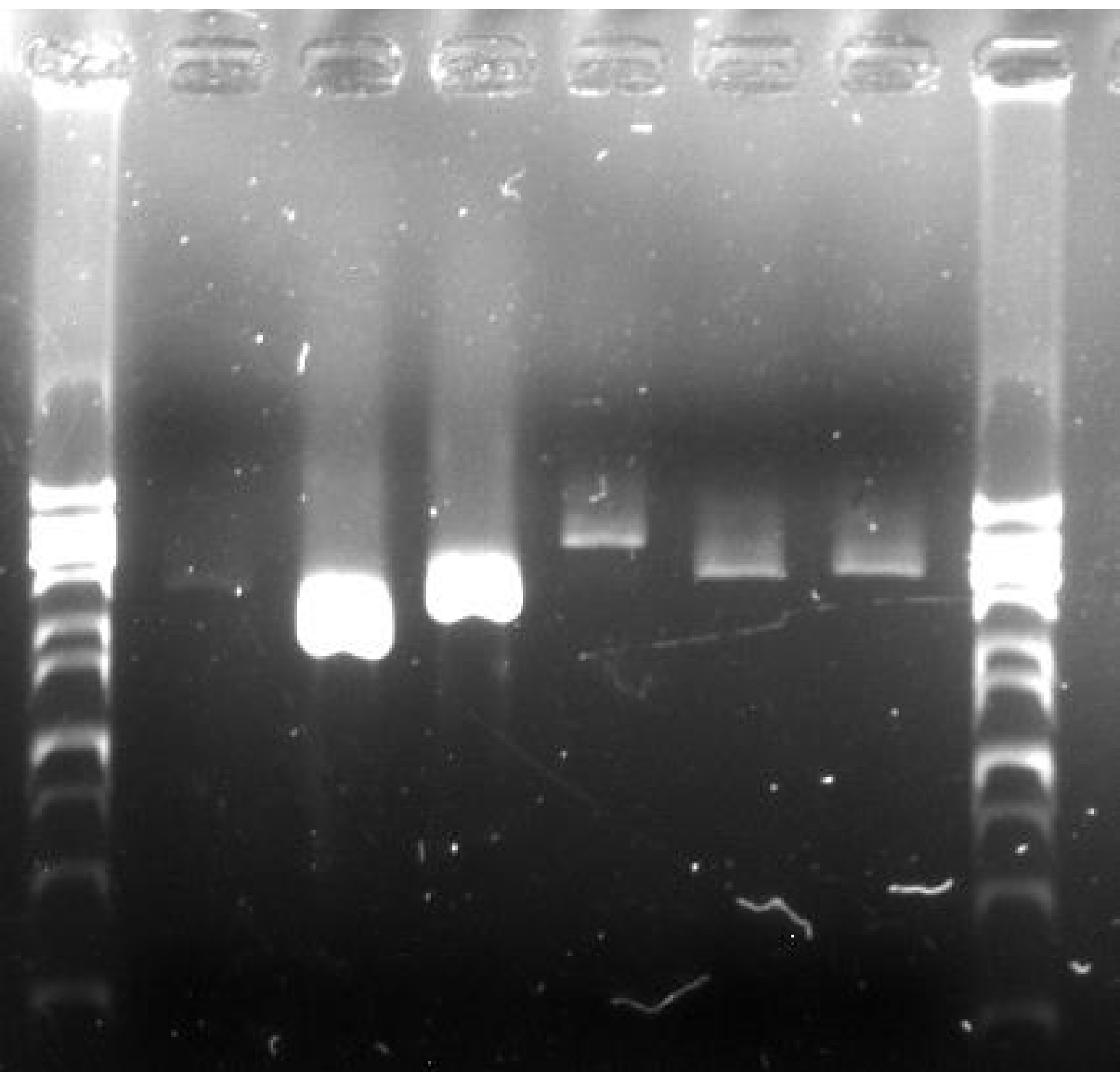

Supplement: S1 Raw images — (PDF) [file pone.0247135.s001.PDF]
