# Supplementary material for: Construction of engineered RuBisCO Kluyveromyces marxianus for a dual microbial bioethanol production system
Source: PLoS One. 2021 Mar 4;16(3):e0247135. doi: 10.1371/journal.pone.0247135 (PMC7932148; doi:10.1371/journal.pone.0247135)
Supplement: Attachment — Submitted filename: Responses-to-Reviewer-2-28-1-2021-SL-Final.docx [file pone.0247135.s002.docx]

Dear Reviewer 2

First of all, we would like to appreciate your constructive comments and suggestions on our work. We have carefully considered the comments and have clearly addressed in the reply. Parts of the manuscript have been modified in response to your suggestion and comments.

Reviewer #2: In this article, *K. marxianus* was used as a host for the transformation of form I and form II RubisCO genes derived from the nonsulfur purple bacterium *R. palustris*. *H. thermocellum* was used to degrade Napier grass and rice straw to generate soluble fermentable sugars. The RuBisCO expression of two engineered *K. marxianus* was analyzed. Growth profiles, ethanol production, and residual reducing glucose/carbon dioxide were also detected and compared in different conditions about WT and engineered strains. This research is very close to industrial applications, but this study is still relatively primary. I’d like to encourage the authors to address the following concerns.

**Major：**

1. Please clarify the definitions of form I and form II RubisCO genes in the Introduction.

**Response:** We added a new paragraph to the Introduction to clarify the definitions of form I and form II RubisCO cassettes as your request. The added paragraph was highlighted using the “Track Changes” function in Microsoft Word. In addition, the Figure 1A and Figure 1C illustrated the form I and form II RubisCO cassette constructions as described in the PGASO method.

“The genome of *R. palustris* consists of two active forms of RuBisCO. The form I RuBisCO includes *cbbL* (large subunit), *cbbS* (small subunit), *cbbR* (transcription regulator) and *cbbRRS* (atypical two-component systems) genes. The *cbbL* and *cbbS* genes are located at the distal end of the cbb_I_ operon and the *cbbRRS* genes are found located between the *cbbR* and *cbbLS* genes. The form II RuBisCO comprises *cbbM* gene which is located at the distal end of the cbb_II_ operon, followed by *cbbA* (fructose 1,6-bisphosphate aldolase), *cbbT* (transketolase), *cbbP* (phosphoribulokinase), and *cbbF* (fructose 1,6/sedoheptulose 1,7-bisphosphatase) genes. In the present study, the *cbbL*, *cbbS*, and *cbbP* genes were chosen to construct the form I RuBisCO cassette and the *cbbM*, *cbbP* genes were selected to construct the form II RuBisCO cassette [14,15]”

2. In Materials and Methods, how to efficiently clone amplified gene fragments into the designated cassettes? And how to insert multiple exogenous genes into *K. marxianus* genome. Please give details.

**Response:** The PGASO method used in this paper relies on the use of overlapping gene fragments and it could be applicable to any host that can undergo homologous recombination. Each gene cassette contains a specific promoter, gene fragment, and a terminator. In the first gene cassette, the 1529 bp sequence identical to the 3’ region sequence of *K. lactis* *Lac4* promoter was used as a promoter, and at the 3’ end of the terminator (TTLac), a 55 bp sequence was designed to be homologous to the 5’ end of the promoter ScPGapDH in the gene cassette 2. The terminator ScTTGap of the gene cassette 2, in turn, had 55 bp overhanging sequence at its 3’ end that was homologous to the 5’ end of the promoter ScPGK in the gene cassette 3, and so on. We hope that the illustration in the Figure 1A and Figure 1C make the PGASO concept clearer to the readers.

In addition, the PGASO method in Materials and Methods was also rewritten to make it easier to understand. This paragraph was highlighted using Track Changes.

**“Multiple-gene cassette construction**

The PGASO method was developed to insert multiple exogenous genes into *K. marxianus* 4G5 genome [21]. Since the PGASO method was fundamentally based on site-specific homologous recombination, overhanging sequences were designed to link at the 5’-upstream sequence of a promoter, and to link at the 3’-downstream sequence of a terminator in order to facilitate an accurate gene cassette assembly into a host genome. Specifically, in the first gene cassette, a 1529 bp sequence, which is identical to the 3’ region of the *K. lactis Lac4* promoter, was used as a promoter in the first gene cassette. In the last gene cassette, a 582 bp sequence, which is homologous to the 5’ region of *K. lactis* *Lac4* promoter, was linked with the 3’ end of the terminator ScTTADHI. It is noteworthy that the identities between *K. marxianus* *Lac4* promoter and *K. lactis* *Lac4* promoter, at the 5’ region and at the 3’ region, are 99.8% and 97.9%, respectively. The other gene cassettes were constructed to contain two parts as follows: (1) a gene sequence, at its 5’ end, linked to a promoter, at its 3’ end, linked to a terminator, and (2) a 55 bp overhanging sequence at the 3’ end of the gene cassette that is homologous to a 5’ end of the promoter of its downstream neighboring gene cassette. Individual gene fragment of *cbbL*, *cbbS*, *cbbM*, and *cbbP* from the genome of *R. palustris* CGA009 were amplified by polymerase chain reaction (PCR). The amplified gene fragments *cbbL*, *cbbS*, *cbbM*, *cbbP* and G418 (kanamycin resistance gene) were then cloned into the predesignated cassette plasmids (Table 1) with their specific independent promoter and terminator as follows: ScPGapDH-*cbbL­*-ScTTGap, KlPGapDH-G418-ScTTGap, KlPADHI-*cbbS*-ScTTGap, ScPADHI-*cbbP*-ScTTADHI, and KlPPGK-*cbbM*-ScTTPGK. Subsequently, gene cassettes for PGASO technique were amplified with KOD plus DNA polymerase kit (TOYOBO Biotech) with specific primers listed in the Table 1. The cloning procedure was performed using *Escherichia coli* strain DH5α cells and Luria-Bertani (LB) medium was used as a culture medium. The antibiotic ampicillin (50 μg/mL) was used to screen the cloned plasmids. The sizes of individual transgenes and gene cassettes were shown in the Table 2. In addition, specific primers for RT-qPCR were designed to confirm the transcriptional activities of the transgenes *cbbS*, *cbbL*, *cbbM*, and *cbbP* in the host cells. Importantly, to verify the sizes and the correct orders of the gene cassettes in their predesignated assemblage in the yeast genome, the combined gene cassettes were also amplified using PCR with the forward primer of the upstream gene cassette and the reverse primer of its adjacent downstream gene cassette (Table 1).

**Table 1. Plasmids and primers used in the PGASO constructions and RT-qPCR experiments.**

| **Plasmid** | **Description** | |
| --- | --- | --- |
| pUC18 | Ampicillin resistant; multicopy plasmid with a ColE1 – type replicon | |
| Cassette 1 pUC18-Kl PLac4 | pUC18 derivative with a 1529 bp portion of *Kluyveromyces lactis Lac4* promoter and *K. lactis* *Lac4* terminator with a 55 bp sequence at 3’ end homologous to *S. cerevisiae GapDH* promoter | |
| Cassette 2 pUC18-Sc PGapDH | pUC18 derivative with *S. cerevisiae GapDH* promoter and TTGap terminator with a 55 bp sequence at 3’ end homologous to *S. cerevisiae PGK* promoter | |
| Cassette 3 pUC18- Sc PPGK | pUC18 derivative with *S. cerevisiae PGK* promoter and TTPGK terminator with a 55 bp sequence at 3’ end homologous to *K. lactis GapDH* promoter | |
| Cassette 4 pUC18- Kl PGapDH | pUC18 derivative with *K. lactis GapDH* promoter *S. cerevisiae GapDH* terminator with a 55 bp overhanging sequence at its 3’ region homologous to *K. lactis PGK* promoter | |
| Cassette 5 pUC18-Kl PPGK | pUC18 derivative with *K. lactis PGK* promoter and *S. cerevisiae* *PGK* terminator with a 55 bp sequence at 3’ end homologous to *K. lactis* *ADHI* promoter | |
| Cassette 6 pUC18-Kl PADHI | pUC18 derivative with *K. lactis* *ADHI* promoter and *S. cerevisiae GapDH* terminator with a 55 bp sequence at 3’ end homologous to *S. cerevisiae ADHI* promoter | |
| Cassette 7 pUC18-Sc PADHI | pUC18 derivative with *S. cerevisiae ADHI* promoter and *ADHI* terminator with a 582 bp sequence at 3’ end homologous to *K. lactis Lac4* promoter | |
| Cassette 2 pUC18-Sc PGapDH-cbbL | Cassette 2 pUC18-Sc PGapDH derivative with *cbbL* | |
| Cassette 6 pUC18-Kl PADHI-cbbS | Cassette 6 pUC18-Kl PADHI derivative with *cbbS* | |
| Cassette 5 pUC18-Kl PPGK-cbbM | Cassette 5 pUC18-Kl PPGK derivative with *cbbM* | |
| Cassette 7 pUC18-Sc PADHI-cbbP | Cassette 7 pUC18-Sc PADHI derivative with *cbbP* | |
| Cassette 4 pUC18- Kl PGapDH-G418 | Cassette4 pUC18- KlPGapDH derivative with *kanR* from Lac4-KanMX cassette [21] | |
| **Cassette** | **Primer** | **Sequence** |
| KlPLac4-KlTTLac4 | KlPLac4-3’ | 5’-CCGCGGGGATCGACTCATAAAATAG-3’ |
|  | KlTTLac4_ScPGapDH | 5’-CTACTATTAATTATTTACGTATTCTTTGAAATG  GCAGTATTGATAATGATAAACTTATACAACATCG  AAGAAGAGTCT-3’ |
| ScPGapDH-*cbbL*-ScTTGapDH | ScPGapDH | 5’-AGTTTATCATTATCAATACTGCCAT-3’ |
|  | ScTTGap_ScPGK | 5’-GGACTCCAGCTTTTCCATTTGCCTTCGCGCTT  GCCTGTACGGTCGTTACCATACTTGGCGGAAAA  AATTCATTTGTAA-3’ |
| ScPPGK-ScTTPGK | ScPGK | 5’- ACTGTAATTGCTTTTAGTTGTGTAT-3’ |
|  | ScTTPGK_KlPGapDH | 5'-GGACTCCAGCTTTTCCATTTGCCTTCGCGCTT  GCCTGTACGGTCGTTACCATACTAAGCTTTTTCG  AAACGCAGAATTTTCG-3’ |
| KlPGapDH-G418-ScTTGapDH | Kl-PGapDH | 5’-AGTATGGTAACGACCGTACAGGCAA-3’ |
|  | ScTTGap_KlPGK | 5'-TACCTTTGATACCATAAAAACAAGCAAATATTCT  TACTTCAAACACACCCGTGGCGGAAAAAATTCATTTGTAAACT-3’ |
| KlPPGK-cbbM-ScTTPGK | KlPGK | 5’-CGGGTGTGTTTGAAGTAAGAATATT -3’ |
|  | ScTTPGK_KlPADHI | 5’-AGGTAAGTATGGTAACGACCGTACAGGCAAG  CGCGAAGGCAAATGGAAAAGCTGGAAGCTTTT  TCGAAACGCAGAATTTT-3’ |
| KlPADHI-cbbS-ScTTGapDH | KlPADHI | 5’-CCAGCTTTTCCATTTGCCTTCGCGCTTGCC-3’ |
|  | ScTTGap_ScPADHI | 5’-GGAATCCCGATGTATGGGTTTGGTTGCCAGA  AAAGAGGAAGTCCATATTGTACACTGGCGGAA  AAAATTCATTTGTAA-3’ |
| ScPADH1-cbbP-ScTTADH1 | ScPADHI | 5’-GTGTACAATATGGACTTCCTCTTTTC-3’ |
|  | ScTTADHI_KlPLac4-5’End | 5’-GAAATTTAGGAATTTTAAACTTG-3’ |
| **Primers used for cloning** | | |
| Phosphorobulokinase (PRK) | cbbP-F’ | 5’-CCGCCTAGGATGTCCCGTAAGCATCCG-3’ |
|  | cbbP-R’ | 5’-CATGCCATGGTTACTTCATGCTTCGTTTGCGGTC-3’ |
| Form I RuBisCO small subunit | cbbS-F’ | 5’-CCGCCTAGGATGCGACTGACCCAAGGC-3’ |
|  | cbbS-R’ | 5’-CATGCCATGGTCAGCCTCCGTAGCGTCG-3’ |
| Form I RuBisCO large subunit | cbbL-F’ | 5’-CCGCCTAGGATGAACGAAGCAGTCACC-3’ |
|  | cbbL-R’ | 5’-CATGCCATGGTTAGACCGAGACCGACGG-3’ |
| Form II RuBisCO | cbbM-F’ | 5’-CCGCCTAGGATGGACCAGTCGAACC-3’ |
|  | cbbM-R’ | 5’-CCTTAATTAATTACGCCGCCTGC-3’ |
| **Primers used for RT-qPCR** | | |
| Phosphorobulokinase (PRK) | r-cbbP-F’ | 5’-GCCGATGAATCGATGGTGG-3’ |
|  | r-cbbP-R’ | 5’-TTCATGCTTCGTTTGCGGTC-3’ |
| Form I RuBisCO small subunit | r-cbbS-F’ | 5’-TGACCCAAGGCTGTTTCTCG-3’ |
|  | r-cbbS-R’ | 5’-TTCAGTTCCATCATCACGCC-3’ |
| Form I RuBisCO large subunit | r-cbbL-F’ | 5’-CCGGCGTGATGGAATACAAG-3’ |
|  | r-cbbL-R’ | 5’-ATACTTCTCCGCCGCAGTCA-3’ |
| Form II RuBisCO | r-cbbM-F’ | 5’-ATGATCGCCTCGTTCCTGAC-3’ |
|  | r-cbbM-R’ | 5’-TTGATGATGGTGCCGACGAT-3’ |
| Actin | 4G5 actin F | 5’-GGGCTTCGGTCAACAAAC-3’ |
|  | 4G5 actin R | 5’-TGGTCGGTATGGGTCAAAAGG-3’ |
| **Primers to confirm gene cassettes order in *K. marxianus* genome** | | |
| Cassette (1 + 2 *cbbL*) | 1-2 F | 5’-CCGCGGGGATCGACTCATAAAATAG-3’ |
|  | 1-2 R | 5’-GGACTCCAGCTTTTCCATTTGCCTTCGCGCTT  GCCTGTACGGTCGTTACCATACTTGGCGGAAAA  AATTCATTTGTAA-3’ |
| Cassette (2 *cbbL* + 3) | 2-3 F | 5’-AGTTTATCATTATCAATACTGCCAT-3’ |
|  | 2-3 R | 5'-GGACTCCAGCTTTTCCATTTGCCTTCGCGCTT  GCCTGTACGGTCGTTACCATACTAAGCTTTTTCG  AAACGCAGAATTTTCG-3’ |
| Cassette (3 + 4 G418) | 3-4 F | 5’- ACTGTAATTGCTTTTAGTTGTGTAT-3’ |
|  | 3-4 R | 5'-TACCTTTGATACCATAAAAACAAGCAAATATTCT  TACTTCAAACACACCCGTGGCGGAAAAAATTCATTTGTAAACT-3’ |
| Cassette (4 G418 + 5) | 4-5 F | 5’-AGTATGGTAACGACCGTACAGGCAA-3’ |
|  | 4-5 R | 5’-AGGTAAGTATGGTAACGACCGTACAGGCAAG  CGCGAAGGCAAATGGAAAAGCTGGAAGCTTTT  TCGAAACGCAGAATTTT-3’ |
| Cassette (5 + 6 *cbbS*) | 5-6 F | 5’-CGGGTGTGTTTGAAGTAAGAATATT -3’ |
|  | 5-6 R | 5’-GGAATCCCGATGTATGGGTTTGGTTGCCAGA  AAAGAGGAAGTCCATATTGTACACTGGCGGAA  AAAATTCATTTGTAA-3’ |
| Cassette (6 *cbbS* + 7 *cbbP*) | 6-7 F | 5’-CCAGCTTTTCCATTTGCCTTCGCGCTTGCC-3’ |
|  | 6-7 R | 5’-GAAATTTAGGAATTTTAAACTTG-3’ |

**Table 2. Size of individual RuBisCO genes, gene cassettes used in the PGASO constructions.**

| **Cassette** | **Promoter-Terminator (bp)** | **RubisCO gene (bp)** | **Promoter-Gene-Terminator (bp)** | **Primers** |
| --- | --- | --- | --- | --- |
| 1 | 2347 | - | KlPLac4- KlTTLac4 (2347) | F-KlPLac4-3’  R-KlTTLac4_ScPGapDH |
| 2 | 1102 | *cbbL* (1457) | ScPGapDH-*cbbL*-ScTTGap (2559) | F-ScPGapDH  R-ScTTGap_ScPGK |
| 3 | 1294 | - | ScPPGK-ScTTPGK (1294) | F-ScPGK  R-ScTTPGK_KlPGapDH |
| 4 | 1145 | G418 (810) | KlPGapDH-G418-ScTTGap (1955) | F-Kl-PGapDH  R-ScTTGap_KlPGK |
| 5 | 1291 | *cbbM* (1386) | KlPPGK-cbbM-ScTTPGK (2677) | F-KlPGK  R-ScTTPGK_KlPADHI |
| 6 | 1301 | *cbbS* (425) | KlPADHI-cbbS-ScTTGap (1726) | F-KlPADHI  R-ScTTGap_ScPADHI |
| 7 | 2068 | *cbbP* (876) | ScPADHI-cbbP-ScTTADHI (2944) | F-ScPADHI  R-ScTTADHI_KlPLac4-5’End |

3. In Figure 2B, there are doubts about the corresponding relationship between the electrophoresis bands and the sizes. E.g.: left 4 and right 2.

**Response:** You are right. This was our mistake when using the wrong gel images. We already carefully revised them and displayed the correct gel images in the Figure 1B and Figure 1D.

4. Please explain the reason why the expression is lower than 1.0 in Figure 2.

**Response:** We used actin as the calibrator gene, thus the gene expression levels of RuBisCO were calculated relatively based on the Ct value of the actin gene. Although the expression level of cbbL was low, the RuBisCO activity, however, could be determined. We already checked the RT-qPCR results again and displayed the new results in the Figure 2.

5. To make the article more logical and clearer, the section “Growth Profiles and Ethanol ….Medium YPD-20” should not be arranged at the end of this manuscript.

**Response:** We already rearranged the section as your request. It is now placed between the RuBisCO assay and the *K. marxianus* cultured in plant biomass hydrolysates part.

**Minor:**
1. Line 103, there is a lack of punctuation at the end of the sentence.

**Response:** We already corrected it.
2. Line 162, “FeSO4.7H20” should be changed to “FeSO4·7H2O”. Line 190 is the same.

**Response:** This is the typo, we already corrected it.

3. Line 121, “ antibiotics” should be changed to “antibiotic”.

**Response:** We already corrected this word as your request.

4. Line 180 and 186, unify the speed unit (rpm/x g). “12.800” should be changed to “12,800”. Please handle similar errors as well.

**Response:** We changed 12.800 to 12,800 based on your comment.

5. The OD of *H. thermocellum* is far greater than *K. marxianus*, can changing temperature overcome growth advantages (60℃-37℃)?

**Response:** We decreased the culture temperature from 60^o^C (for *H. thermocellum*) to 37^o^C for *K. marxianus* after *H. thermocellum* entered its decline phase. In other word, after 6 days of culture, *H. thermocellum* finished the biomass hydrolysis step and only maintained its low activity. Subsequently, the decrease in culture temperature to 37^o^C was suitable for the *K. marxianus* growth. By this way, we tried to prevent the potent competition between two species in the co-culturing system.

6. “After 144 h, the culture stopped producing H_2_ and CO_2_, indicating the end of metabolic activities.” The measurement of hydrogen is not mentioned in this paper. Please explain how to know the H2 production ceased.

**Response:** We measured H_2_ production at the same time with CO_2_ using the same GC protocol. We already modified this part to make it clearer.

7. The size of letters should be constant in Figures 4 and 7. Especially for Figure 7, some words are too small to read.

**Response:** We already redrew all the graphics with bigger font size as your request

8. Ethanol concentration should use “g/L” as a unit.

**Response:** We agree with the reviewer that ethanol concentration should be displayed as g/L, but we also think mg/L is acceptable. Therefore, we decided to use this unit consistently in this paper.

9. Is there any special reason to choose Box plot for ethanol production? Why not use a line plot like residue sugar. Actually, sugar and ethanol can be put into one figure.

**Response:** In the case of residual sugar, we measured sugar concentrations at different timepoints from the beginning of the culture (0 h), intermediate timepoints to the end of the process (24 h, 48 h, and 72 h) to observe the patterns of sugar consumption by *K. marxianus*. Therefore, line plot is a suitable way to describe the data. Ethanol measurement, in contrast, was only carried out at the end of the fermentation and the ANOVA was calculated based on the raw data collected from biological triplicates. In addition, boxplot is a useful way to show the contribution of data, their central values, and their variability. The use of boxplot is preferable to the barplot (with mean and standard deviation) when the data are not strictly symmetrically distributed.

Dear Reviewer 2

We hope that our responses and correction are satisfactory to you, and that it is now suitable for publication in PLOS ONE Journal.

Dr. Chieh-Chen Huang

On behalf of all authors
